# Association of Circulating Tumor Cells with Inflammatory and Biomarkers in the Blood of Patients with Metastatic Castration-Resistant Prostate Cancer

**DOI:** 10.3390/life11070664

**Published:** 2021-07-06

**Authors:** Gerit Theil, Carlotta Lindner, Joanna Bialek, Paolo Fornara

**Affiliations:** Medical Faculty of Martin Luther University Halle-Wittenberg, University Clinic and Outpatient Clinic for Urology, 06120 Halle (Saale), Germany; Carlotta.lindner@uk-halle.de (C.L.); joanna.bialek@uk-halle.de (J.B.); paolo.fornara@uk-halle.de (P.F.)

**Keywords:** biomarker, circulating tumor cells, prostate cancer

## Abstract

The identification of specific biomarkers that recognize the functional drivers of heterogeneity in prostate cancer (PCa) and personalized treatment remain challenging in systemic medicine. Liquid biopsy allows for the detection and analysis of personalized predictive biomarkers in single blood samples and specifies the current stage of cancer. The aim of our preliminary study was to investigate the association between an elevated circulating tumor cell (CTC) count and the levels of inflammatory factors (IL-6 and IL-8) and biomarkers (DKK-1, PSA, sHER2, and CD44) in patients with metastasized castration-resistant PCa (mCPRC) under chemotherapy and those with localized PCa. Such an association could be used as a component of cancer progression monitoring. We compared the sensitivity and specificity of two CTC isolation platforms. Twenty-eight patients (12 mCRPC and 16 localized PCa patients) were enrolled. Over the study period, the CTC detection rates were 84% with CellCollector^®^ and 73.5% with CellSearch^®^ System in mCPRC patients. The CTC counts determined by the CellSearch^®^ System (CTC_CS) were correlated significantly with the DKK-1, sHER-2, and PSA concentrations in mCRPC patients. The CTC counts captured by CellCollector^®^ demonstrated no significant association with the concentrations of the tested blood-based biomarkers. The CTC_CS count (AUC = 0.9 (95% CI: 0.72–1.0)) and the PSA level (AUC = 0.95 (95% CI: 0.83–1.0)) presented approximately the same sensitivity and specificity for the overall survival of mCRPC patients. For better personalized characterization, further research on CTC phenotyping and their interactions with tumor-associated blood-released factors is needed.

## 1. Introduction

Prostate cancer (PCa) is the fifth leading cause of cancer-related death worldwide [1]. The incidence increases with each decade of age, and thus, 59% of men over 79 years of age have PCa [2]. In an aging population, more PCa would be diagnosed. Furthermore, in men aged 75 years and older, the incidence of regional- and distant-stage disease increased from 2013 to 2016 [3]. For 28% of patients with distant metastasis, the estimated survival rate is approximately 5 years [4]. The majority of these patients have multifocal metastatic sites, such as bone and lymph nodes (particularly vertebrae and pelvis) [5]. Moreover, oligometastatic PCa has distinct biological states and harbors different mutations, which result in heterogeneous phenotypes. Metastatic progression requires certain characteristics of cancer cells, such as plasticity, motility, and colonization, as well as systemic physiological conditions, such as inflammation, which are drivers of metastasis and therapeutic resistance in PCa [6]. Drug resistance is a dynamic process in tumor cells, which includes molecular events such as genome modification and the regulation of diverse transcriptional states. Additionally, cancer cells undergo phenotype acquisition in the process of cellular rewiring [7]. In the last decade, innovations in treatments and combination therapeutic strategies have been developed and have contributed to the therapeutic armamentarium, improving the outcomes from metastatic PCa [8,9].

Nevertheless, the determination of the optimal personalized drug sequence to minimize possible therapeutic resistance remains a challenge [10]. Therefore, personalized biomarkers of these characteristics are needed to determine treatment responses and facilitate decisions on the selection of agents.

Classic clinical factors, such as the blood levels of prostate-specific antigen (PSA), and pathological factors, such as Gleason grading and tumor, node, and metastasis (TNM) staging, are well-known prognostic markers in PCa [11]. However, these methods are often insufficient for accurate risk stratification, and do not adequately describe the metastatic process. One possibility is liquid biopsy, which includes (among others) circulating tumor cells (CTCs). CTCs detach from primary or metastatic tumors to enter the bloodstream, and a small CTC population has the ability to metastasize to multiple organs [12]. They provide characteristics of the current stage of the tumor or potential metastasis and allow for the real-time monitoring of therapeutic responses. CTCs’ interplay with blood components is important for their survival and metastatic characteristics [13,14]. They may interact with neutrophils, platelets, leukocytes, monocytes, and macrophages in the circulation, which protect the CTCs from rapid clearance by natural killer cells and the physical shear stress of blood flow. These interactions promote the survival and extravasation of CTCs at distant sites [13].

The cytokines interleukin 8 (IL-8) and IL-6 are associated with inflammation contributing to PCa and progression to treatment resistance. IL-8 is secreted by monocytes, neutrophils, and endothelial cells. Its signaling in PCa cells is involved in regulating the transcriptional activity of the androgen receptor (AR), and substantiates the transition to an androgen independent proliferation of prostate cancer cells [15]. Furthermore, IL-8 overexpression by tumor cells is often induced in response to chemotherapeutic treatment and may be important in the tumor microenvironment [16,17].

IL-6 stimulates proliferation, promotes angiogenesis, and inhibits apoptosis of PCa cells and other tumor cells. These activities are due to the interaction of IL-6 with multiple signaling pathways, such as the Janus tyrosine family kinase (JAK)-signal transducer and activator of transcription (STAT) pathway and the extracellular signal-regulated kinase 1 and 2 (ERK1/2)-mitogen activated protein kinase (MAPK) pathway [18]. Additionally, IL-6 has been identified as a nonsteroidal compound of AR activation (N-terminus of AR), which is different from ligand activation [19,20]. IL-6 is also known to induce human epidermal growth factor receptor-2 (HER2) signaling through the MAPK pathways [21]. HER2 belongs to the epidermal growth receptor family, which regulates processes such as cell differentiation, migration, and survival. The activation of HER2 results in ligand-independence over homodimerization, heterodimerization with other receptors of the HER family, or proteolytic cleavage of the extracellular domain (sHER2 ECD) [22]. HER2 signaling promotes AR signaling through androgen ligand-independent mechanisms and supports the development of castration-resistant PCa (CRPC) [23,24]. Ma et al. [25] demonstrated that CD44 interacted with HER2 promotes DNA damage repair and radioresistance. Moreover, CD44 expression in cancer cells promotes bone metastases by enhancing tumorigenicity, cell migration, and progression [26,27].

CRPC patients mostly have bone metastasis, which results in skeletal-related events such as pathological fractures. Osteoblast function is dependent on Wnt signaling, controlled by the Wnt inhibitors sclerostin and Dickkopf1 (DKK-1) [28]. Furthermore, DKK-1 expression in tumor cells activated Wnt/β-catenin signaling and demonstrated an interaction with AR signaling [29].

The aim of our preliminary study was to investigate the association of an elevated CTC count with inflammatory molecules (IL-6 and IL-8) and biomarkers (DKK-1, sHER2, and CD44) in patients with metastasized CPRC (mCPRC) under chemotherapy and localized PCa (PCa-l). Such an association could be used as a component of cancer progression monitoring.

## 2. Materials and Methods

### 2.1. Patient Cohorts

This is a retrospective analysis of a subpopulation of a prospectively planned clinical trial in the University Clinic and Outpatient Clinic for Urology, Medical Faculty of Martin Luther University Halle-Wittenberg [30]. All of the patients provided written informed consent and were enrolled in the study. This included blood sampling (4.5 mL serum) for future research. The protocol was approved by the medical faculty ethics committee of Martin Luther University Halle-Wittenberg (number of ethical approval: FSMW EPCAM-Prostata-M00, 2012-65). The men enrolled in the first group were patients with histologically confirmed prostate adenocarcinoma with progressive disease, despite castration levels of serum testosterone (<50 ng/dL). Only two of the patients achieved the castration-resistant stage in the second month of the study. All 12 patients were examined every month for 6 months, followed by visits in the 8th and 12th months, for a total of eight visits. CTC evaluation with CellCollector^®^ and the CellSearch^®^ System and blood sampling for additional biomarker analysis were taken before starting the chemotherapy or the bone-targeted therapy. The second group included patients with confirmed prostate adenocarcinoma, who had opted for radical prostatectomy (RP) in the observation period and were assessed three times within 12 months. The first visit was before the prostate removal. The next visits were 6 and 12 months after surgery.

### 2.2. Sample Collection

Additionally, 9 mL of blood serum was collected for the determination of the levels of PSA, C-reactive protein (CRP), and testosterone and for Luminex analysis. Samples were collected at each visit. The serum was processed within one hour after collection through centrifugation at 1300× *g* for 10 min. The samples were stored at −80 °C.

### 2.3. CTC Isolation

We used two different methods for CTC isolation, the CellSearch^®^ System (Silicon Biosystem, Menarini, Florence, Italy) and CellCollector^®^ (GILUPI GmbH, Potsdam, Germany), at matched times. Both systems used an epithelial cell adhesion molecule (EpCAM) antibody to capture the CTCs, as previously described [30,31].

CellCollector^®^, a medical wire, was carefully inserted into the patient’s cubital vein via a 20G peripheral venous catheter until the tip of the wire (2 cm) was in the bloodstream of the vein. After 30 min, the wire was pulled out of the vein. In the first step, the captured cells were fixed with 100% acetone for 10 min at room temperature, blocked with 3% bovine serum albumin/PBS for 30 min, and then prepared for characterization.

For the CellSearch^®^ System analysis, 7.5 mL of blood was collected in CellSave^®^ Preservative Tubes. These samples remained stable for 96 h at room temperature and were sent overnight to the Department of Tumor Biology, University Medical Center Hamburg-Eppendorf, Hamburg, Germany.

### 2.4. CTC Characterization

The matched pair analysis requires the same identification criteria as that of the CTCs. The captured cells were stained with fluorescein isothiocyanate (FITC)-labeled antibodies against cytokeratin 8, 18, and 19 (eBioscience, Abcam) for the detection of epidermal cancer cells in the blood. CD45 staining (anti-CD45-A647, Exbio) was performed to exclude leucocytes. Additionally, the cells’ nuclei were stained with Hoechst 33342. Cells were defined as CTCs when they met the following cytology-based FDA definition: (i) size ≥4 µm, (ii) visible cytoplasm, (iii) high nuclear/cytoplasm ratio, and (iv) positive fluorescent staining, as described above [32,33].

The images were digitally processed with ImageJ software by altering the contrast and brightness in accordance with Nature Publishing Guidelines [34].

### 2.5. Detection of Circulating Biomarkers

The serum levels of sHER2, IL-8, IL-6, DKK-1, and CD44 were simultaneously determined by a custom-made configuration of the Luminex Screening Human Magnetic Assay (R&D Systems). The assays were conducted following the manufacturer’s instructions and were performed on a Luminex 100^TM^ Qiagen GmbH system (Hilden, Germany). All of the serum samples required a two-fold dilution in calibration diluent. For the analysis, we used a 96-well flat bottom microplate. The measurements for each sample were performed in duplicate, and the average of the two measurements was used. Limits of quantification were determined using the lowest or highest standard point and a percent CV (%CV = 100 × standard deviation/average) of less than 20%. PSA and CRP were determined with Immulite 100 (Siemens Healthcare Diagnostics GmbH, Eschborn, Germany), according to the manufacturer’s instructions.

### 2.6. Statistical Analysis

All of the determined blood-based biomarkers or metabolites were normalized. The values obtained at the first visits were defined as 100%. The relative secretion values are shown in box plots with medians and interquartile ranges (IQRs). Whiskers represent the minimum and maximum values. Furthermore, all of the data were tested for normal distribution using the Shapiro−Wilk test, and the parameters are presented as the median ± range.

Finally, for the identification of possible correlations between the different markers for the different study groups, Spearman’s rank correlation coefficient (r_s_) was determined and is represented in a heatmap. The reported p-values are two-sided, and ≤0.05 was considered significant. The accuracy of the selected biomarker levels was evaluated by receiver operating characteristic (ROC) analysis. For this analysis, we used no cut-offs, but the median was six visits performed for 24 months survival. The optimal cut-off for the Kaplan−Meier analysis of PSA based on the ROC curve was calculated by the largest value of the formula, sensitivity + specificity − 1, from the median PSA level for every mCRPC patient (likelihood ratio). The mean CTC count was determined based on the CTC counts of visits 1–8 (V1–V8). Kaplan−Meier analysis was used to analyze the overall survival (OS) depending on the mean CTC count. The survival estimates in different groups were compared using the log-rank (Mantel−Cox) test. All of the statistical analyses were performed using GraphPad Prism software versions 7 and 9.

## 3. Results

### 3.1. Study Design and Patient Data

A total of 28 patients (12 mCRPC patients and16 PCa-l patients) were enrolled in the analysis. All of the study-related applications were identical in the groups. Age (*p* = 0.09) and body mass index (*p* = 0.18) were not significantly different between the groups (Mann-Whitney test). The median age was 68.5 years in the mCRPC patients and 63 years in the PCa-l patients. The median BMI was 27.5 in the mCRPC patients and 29.7 in the PCa-l patients. The Gleason score was significantly different (*p* < 0.0001) between the PCa-l and mCRPC groups (Mann-Whitney test). Ten patients (83.3%) received docetaxel in combination with prednisone as the first-line treatment for mCRPC, and three (25%) received cabazitaxel (one patient switched in the study period from docetaxtel to cabazitaxel) in response to resistance to docetaxel. The PCa-l patients were treated after the first visit with laparoscopic RP (82.3%) or high-intensity focused ultrasound (HIFU) (11.76%). The other baseline characteristics are summarized in Table 1.

### 3.2. Assessment of Different Serum and Blood Biomarkers

**Figure 1:**
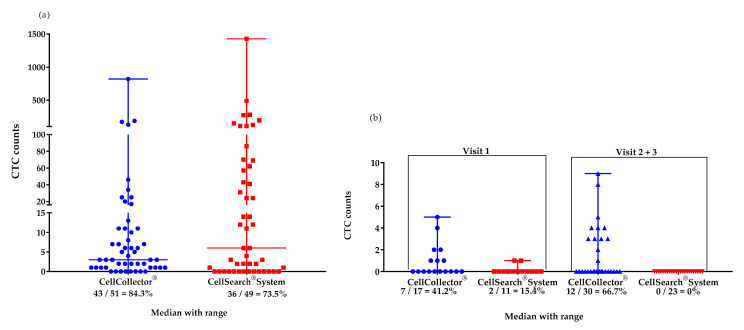
Median (range) values of CTCs isolated with the CellCollector^®^ and CellSearch^®^ Systems. (**a**) mCRPC patients (*n* = 12) in the study period of 12 months: CellCollector^®^, 4 CTCs (0–820), and CellSearch^®^ System, 8.5 CTCs (0–1428); (**b**) PCa-l patients (*n* = 16) at visit 1 (before surgical removal): CellCollector^®^, 0 CTCs (0-5), and CellSearch^®^ System, 0 CTCs (0–1) and at visits 2 and 3 (6 and 12 months after removal of prostate, respectively): CellCollector^®^, 0 CTCs (0–9), and CellSearch^®^ System, 0 CTCs (0).

We isolated CTCs with two different EpCAM-based systems from the mCPRC (*n* = 12) and PCa-l (*n* = 16) patients. Over the study period, the CTC detection rates were 84% with CellCollector^®^ (CTC_CC) and 73.5% with the CellSearch^®^ system (CTC_CS) in the mCPRC patients. Furthermore, the CTC-median in the mCRPC patients did not differ significantly (*p* = 0.29) between the two isolation platforms. A median of 4 CTCs (range 0–820) was captured by CellCollector^®,^ and 8.5 CTCs (range 0-1428) by the CellSearch^®^ system (Figure 1a). The baseline CTC count was zero in one mCPRC patient with CellCollector^®^ and in three patients with the CellSearch^®^ system. At the first visit, seven patients (58.8%) had <5 CTCs and three (25%) had ≥5 CTCs, as determined with CellCollector^®^. When the CellSearch^®^ system was used, one (8.3%) patient had <5 CTCs and nine (75%) patients had ≥5 CTCs.

The PCa-l group had a median of 0 CTCs detected with both platforms at the first visit; 0–5 CTCs were achieved with CellCollector^®^ and 0–1 CTCs were achieved with the CellSearch^®^ system. In addition, in the cured patients, 0 CTCs were detected using the CellSearch^®^ system. However, CellCollector^®^ captured a median of 0 CTCs with a range of 0–9 at visits 6 and 12 months after RP (Figure 1b).

Biomarkers were measured until visit 6 (sixth month) in the study period. Unfortunately, data from visits 7 and 8 could not be included in the analysis because of an insufficient sample size. The serum levels of sHER2, IL-8, IL-6, Dkk-1, and CD44 did not show significant differences between the PCa-l and mCRPC patients (Table 2). The median levels of DKK-1 (4625 pg/mL), IL-6 (11.7 pg/mL), and IL-8 (20 pg/mL) in the mCRPC patients were higher than those in the PCa-l patients (3939 pg/mL, 5.6 pg/mL, and 10.8 pg/mL, respectively). Interestingly, the CD44 level in the mCRPC patients was the lowest in the study population. Moreover, the sHER level demonstrated a decreased concentration over six months in the mCRPC group. The median secretion levels were 3.3 ng/mL in the PCa-l group at visit 1 and 3.5 ng/mL at visits 2 and 3. The mCRPC patients had a median concentration of 3.3 ng/mL, which was approximately equal to the concentrations in the localized cancer stage groups. Interestingly, the sHER concentration had the widest range of 0.3–16.64 ng/mL in the mCRPC group. Significant differences were found for the PSA level (*p* < 0.001), CRP level (*p* = 0.03), and CTC count (*p* < 0.001) between the groups (Table 2).

**Figure 2:**
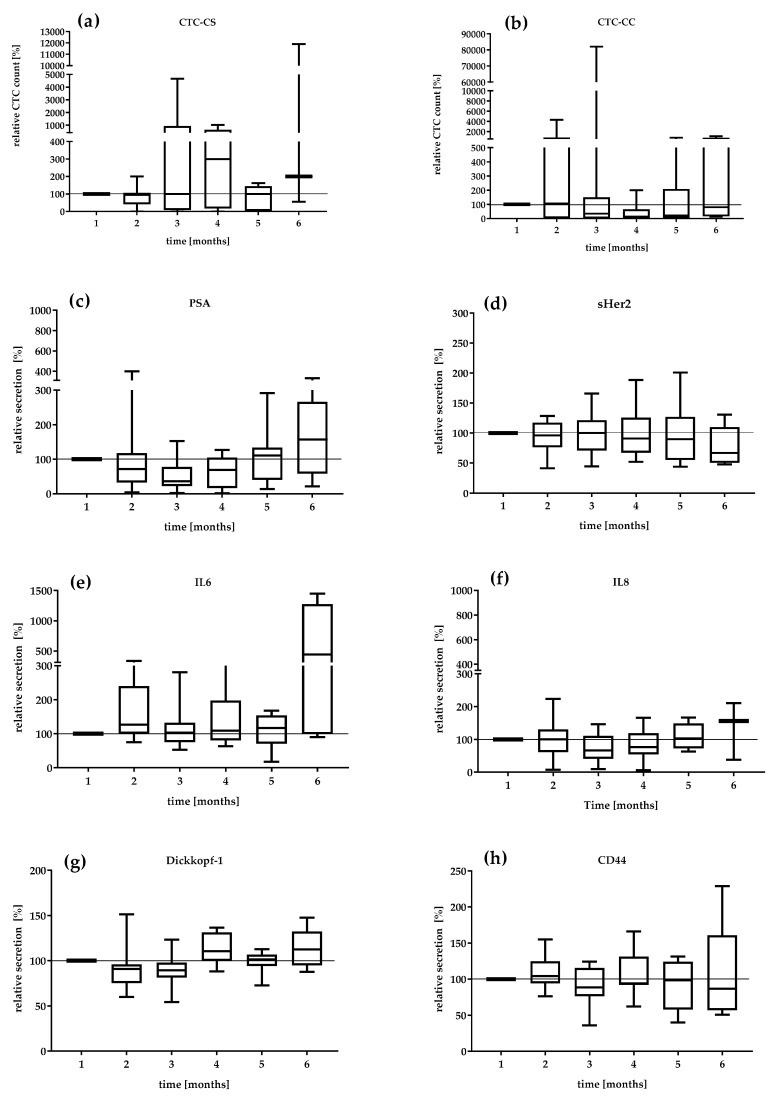
Relative value in percent of (**a**) CTC_CS count, (**b**) CTC_CC count, (**c**) PSA, (**d**) sHER2, (**e**) IL-6, (**f**) IL-8, (**g**) DKK-1, and (**h**) CD44 in the mCRPC patients during 6 months. The median relative secretion with minimum and maximum values. The value of the first visit was defined as 100%.

We investigated the serial secretion of the biomarkers in the treatment follow-up at 6 months in the mCRPC patients (Figure 2). The first values were defined as 100%. The median relative CTC_SC count and the median relative secretion of PSA, IL-6, and IL-8 during the settlement period were the most dynamic markers (Figure 2a,c,e). The CTC count continually changed from 95 to 300% from visit 2 to visit 4. In contrast, the relative CTC_CC counts demonstrated a decreasing level during the period of analysis. The lowest relative CTC_CC count was reduced by 14% at visit 4 (Figure 2b). The median PSA level also showed variations with a range of 36% at visit 3 and 157% at visit 6. The DKK-1 protein showed a relatively constant secretion of 90.1–112.5%. IL-6 secretion remained relatively constant in the range of 126–117% until the fifth month. Interestingly, IL-6 secretion increased 440% in the 6th month. sHER-2 showed variations in a range of 96–66.8%, which revealed a continuous decrease in concentration under therapy. The serial change in IL-8 secretion demonstrated a variation of 66% in the third month to 156% in the 6th month. CD44 secretion was relatively constant over the observation period (104–86%).

**Figure 3:**
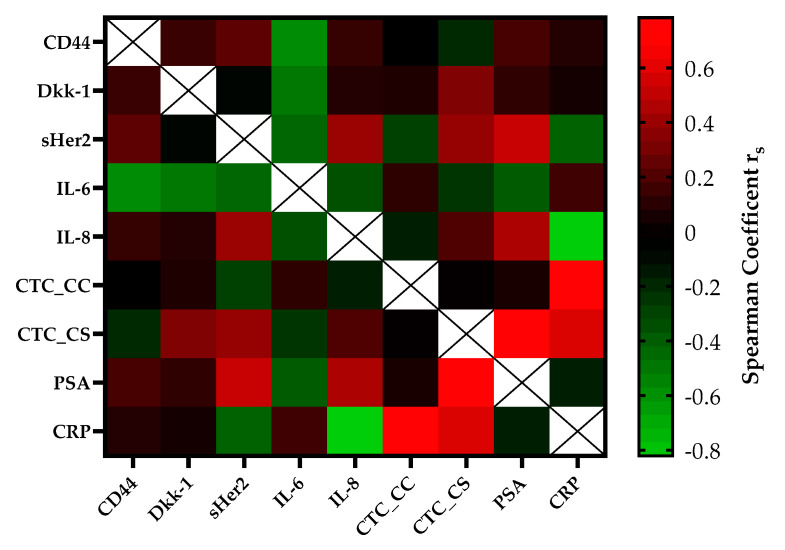
The heatmap of the correlation coefficients (Spearman) among biomarkers of the mCRPC patients. The color-coded correlation is on the left, where red demonstrates a strong positive correlation and light green indicates a strong negative correlation.

The correlation of serial CTC secretion between the serial secretion of biomarkers and inflammatory markers is shown in Figure 3. CTC counts determined with the CellSearch^®^ system (CTC_CS) were moderately positively correlated with the concentrations of DKK-1 (r_s_ = 0.35, *p* = 0.01) and sHER-2 (r_s_ = 0.41, *p* = 0.004) in the mCRPC patients. A strong correlation was found between the CTC_CS count and the PSA concentration (r_s_ = 0.75, *p* ≤ 0.0001) and the CTC counts of both platforms (r_s_ = 0.78, *p* = 0.03). Within regard to the CTC count captured by CellCollector^®^ (CTC_CC), no significant association was observed with the concentrations of the other blood-based biomarkers. The CRP concentration was strongly positively, but not significantly correlated with the CTC count (CTC_CS r_s_ = 0.60, *p* = 0.4; CTC___CC r_s_ = 0.78, *p* = 0.078). Interestingly, we demonstrated a good correlation between PSA and sHer2 levels (r_s_ = 0.55, *p* ≤ 0.0001) and PSA and IL-8 levels (r_s_ = 0.47, *p* ≤ 0.0001) in our cohort. For IL-6, a good negative correlation was observed with DKK-1 (r_s_ = −0.45) and CD44 (r_s_ = −0.54).

In the PCa-l patient group, no significant correlation was found between the CTC counts of either platform and the biomarker levels; however, the levels of markers prior to prostate removal were correlated (Supplementary Figure S1).

**Figure 4:**
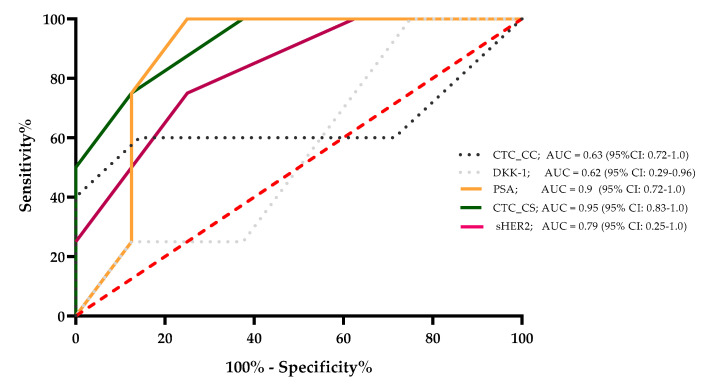
A survival ROC curve was plotted to evaluate the sensitivity, specificity, and AUC of serum concentrations of sHer2, PSA, DKK-1, CTC_CC, and CTC_CS and the 24-month survival.

The sensitivity, specificity, and area under the curve (AUC) value were determined for CTC_CC, DKK-1, PSA, CTC_CS, and sHER2, which were correlated significantly with the CTC_SC count. The results demonstrated that for a survival time of 24 months, the AUC values of these markers were 0.63, 0.62, 0.9, 0.95, and 0.79, respectively. The PSA level and the CTC_CS showed the strongest ability to predict survival for 24 months for the mCRPC patients. These results were calculated with the 6-month median level of the evaluated markers for every single mCRPC patient (Figure 4).

### 3.3. The OS Value of CTC Count Versus PSA Level

We reached a follow-up time of 5 years in the study population, and compared the prognostic value of the median CTC count and the PSA concentration. In our analyses, we used the established CTC cutoff values of <5 or ≥5 CTCs [32]. For the PSA level, we used the estimated cutoff value of 53 ng/mL, which was calculated for the mCRPC patients in our study. In this study, the positive likelihood ratio was 8.0 (sensitivity 100%, specificity 87.5) for PSA cutoff values of 53 ng/mL.

**Figure 5:**
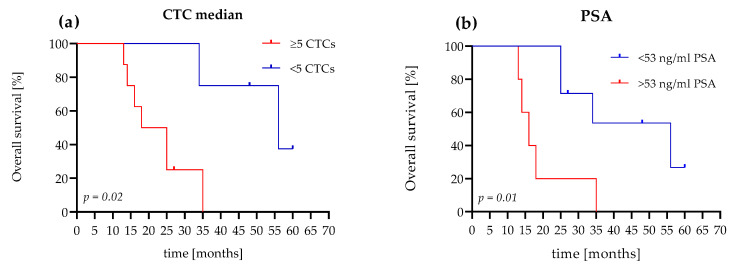
Comparison of Kaplan–Meier curves for OS according to the CTC count and PSA level of the mCRPC patients. (**a**) The patient shows <5 CTCs and a ≥5 CTC difference in OS (56 months versus 21.5 months (HR 4.6, 95% CI, 1.2–17)). (**b**) The patient shows a PSA level <53 ng/mL and a ≥53 ng/mL difference in OS (56 months versus 16 months (HR 4.4, 95% CI, 0.9–21)).

Patients (75%) with evaluated CTC counts of <5 cells survived 34 months, with a median of 56 months. Patients (75%) with an evaluated CTC count of ≥5 cells survived 14 months, with a median survival time of 21.5 months. The hazard ratio (HR), referring to <5 or ≥5 CTCs, was 4.6 (95% confidence interval (CI): 1.2–17.03; Figure 5a).

In comparison, with a PSA level <53 ng/mL, 71.5% of the patients survived 25 months with a median of 56 months. Patients (60%) with PSA levels >53 ng/mL survived 14 months and had a median survival time of 16 months. The HR, referred to as <53 ng/mL PSA or >53 ng/mL PSA, was 4.4 (95% CI: 0.9–21; Figure 5b).

## 4. Discussion

We analyzed the association between inflammatory markers and different biomarkers under therapy in a cohort of patients with PCa-l and mCRPC. Moreover, we compared two CTC isolation platforms for their sensitivity and specificity. There are many CTC isolation platforms; however, all of them have disadvantages and advantages [35,36]. We used CellCollector^®^, an in vivo CTC isolation system [30,37], and the FDA-approved CellSearch^®^ system [32]. Similarly, in both platforms, CTCs were captured using antibodies against the EpCAM protein and were further characterized. The CellSearch^®^ system required a blood sample of 7.5 mL, while CellCollector^®^ required a larger volume. The CellSearch^®^ system detected a higher CTC count in the mCRPC group, although the detection rate of CellCollector^®^ was 84% compared with 73.5% of the CellSearch^®^ system. Nevertheless, a range of 0–9 CTCs detected using CellCollector^®^ in PCa-l patients compared with a range of 0–1 CTCs detected using the CellSearch^®^ system. These results indicated that CellCollector^®^ might be more useful than the CellSearch^®^ system in nonmetastatic PCa patients because of the higher CTC detection rate. A possible reason for the different results could be the different EpCAM antibodies with differences in the affinity to the EpCam molecule. Furthermore, the veins in localized PCa patients are sometimes better for the in vivo application of the CellCollector^®^ as in mCRPC patients. Even if the number of detected CTCs in indolent localized patients is low and their clinical utility remains unclear, their better specified molecular characterization would be crucial for clinical application. Chen et al. [38] further assessed high-risk nonmetastatic PCa patients and described CellCollector^®^ as an efficient CTC technology for monitoring cancer relapse in localized PCa, as well as for monitoring of the treatment response.

The CTC counts obtained with CellCollector could also be tested in metastatic castration-sensitive prostate cancer patients (mCSPC) as biomarkers for evaluation of the treatment with androgen-receptor-axis-targeted (ARAT) therapy compared with docetaxel to improve the outcome in mCSPC patients [39,40].

However, in a comparison of different CTC platforms (CellCollector^®^, dual fluoro-EPISPOTPSA/FGF2, and the CellSearch^®^ system), the CellSearch^®^ system was the most accurate predictor of metastatic PCa (AUC 0.76, 95% CI: 0.631–0.908) [41]. Our ROC analysis showed an AUC of 0.95 (95% CI: 0.83–1.0) for the CellSearch^®^ system, which confirmed the high sensitivity and specificity of this system. The PSA level, a classic marker in blood-based therapeutic monitoring of advanced PCa patients, demonstrated a comparatively high sensitivity and specificity with an AUC of 0.90 (95% CI: 0.72–1.0) in our mCRPC patient cohort (Figure 4). Interestingly, our results demonstrated a good correlation between the PSA level and the CTC count determined with the CellSearch^®^ System. CTCs are prognostic parameters in mCRPC patients, but are usually independent of the PSA levels [32,42,43,44]. In our Kaplan–Meier OS analysis, a CTC count of ≥5 cells and >53 ng/mL PSA showed nearly identical HRs (CTC count HR = 4.6, *p* = 0.02 and PSA level HR = 4.4, *p* = 0.01). Our data showed that the CTC count and the PSA value in our cohort of mCRPC patients presented almost identical prognostic values. Nevertheless, CTCs can provide additional cancer-specific characteristics at the protein, mRNA, and DNA levels [35].

Furthermore, we found elevated serum levels of sHER2, DKK-1, IL-6, and IL-8 in the mCRPC patients and the PCa-l patients and found no significant difference between the groups (Table 2). Moreover, all of the analyzed markers were actively or passively involved in the bypassing of the AR signaling and might indicate active signaling in the blood. These factors may also influence the ability of CTCs to enhance inflammatory factors and biomarker release in blood circulation for possible crosstalk with cells.

Moreover, we found that the median DKK-1 serum level of 4625 pg/mL in the mCRPC patients was slightly increased compared with that in the PCa-l patients (3939 pg/mL), which may contribute to the development of osteoblastic metastasis. In addition, the higher DKK-1 concentration could indicate a possible switch in phenotype to the osteoblastic metastasis type [45]. In the serial measurements, a variation of 90.1–112.5% of DKK-1 was observed (Figure 3g). Interestingly, in the sixth month of systemic therapy, an increase of 112% was observed, as well as increases in the levels of PSA (157%), IL-6 (440%), CTC_CS (200%), and IL-8 (156%), which was consistent with the docetaxel treatment interruption (Figure 3). The doubling of the median CTC count suggests active cancer communication or micrometastatic progression. The increased serum level of DKK-1 could be due to the zoledronic acid treatment of the mCRPC patients, as shown by Thiele et al. [46] in an analysis of serum samples at different PCa stages. Our mCRPC cohort was under zoledronic acid treatment.

However, a good negative correlation of −0.45 (*p* < 0.0001) was demonstrated for DKK-1 and IL-6. This effect was described in inflamed joints of rheumatoid arthritis [47]. The median IL-6 and IL-8 concentrations in the serum of the mCRPC patients were substantially increased compared with those in the PCa-l group (Table 2). Culig [48] postulated in his review that serum IL-6 can act as an attractant for tumor cells and is linked to aggressive tumors. The IL-6 concentration of our cohort (11.7 pg/mL in the mCRPC group) was similar to that of the cohort of Nakashima et al. [49], and higher than 7 pg/mL. The increase of 440% in the sixth month of treatment could indicate active signaling pathways in PCa. Our results confirmed the findings from these studies, which concluded that higher IL-6 serum levels were correlated with the tumor stage and were inversely correlated with tumor survival and therapeutic response [18,48]. In the monitoring of mCRPC patients, the IL-8 level increased to 156% (visit at 6 months) compared with the baseline level (100%). Maynard et al. [50] reported that the high expression of IL-8 in the tumor microenvironment is associated with aggressive PCa and with the loss of the AR. Analysis of the IL-8 serum level of PCa-l patients found no correlation with diagnosis and aggressiveness [51]. We also detected lower IL-6 and IL-8 concentrations in the serum of the PCa-l group. In the mCRPC group, we could not demonstrate any significant correlation of interleukins 6 and 8 with the CTC count. One possible explanation could be the CTC status in the blood circulation and current tumor stage, which need to be explored in further studies. It is known that CTCs undergo a phenotype switch from epithelial to mesenchymal transition (EMT), and present a mesenchymal status [52]. Patients with newly diagnosed metastatic castration-sensitive PCa and positive for mesenchymal CTCs show a decline in resistance to androgen deprivation therapy compared with patients who are negative for EMT CTCs [53].

Interestingly the serum level of the sHER2 showed a significant (*p* < 0.001) moderate (r_s_ = 0.41) correlation with the CTC_SC count. Although we could detect sHER2 in the serum, the median concentration was equal in our groups, but the range (0.83–16.46 ng/mL) in the mCRPC group was much wider. This finding suggests that CTCs in the blood circulation express HER2, and that HER2 signaling is activated through the cleavage of sHER2 (ECD). The single patient profile shows an increasing sHER2 concentration in the fifth and sixth months (data not shown), which is consistent with chemotherapy interruption. Josefsson et al. [54] demonstrated a high correlation between HER2 expression in CTCs and metastatic samples, and emphasized the potential for CTC phenotyping for individualized therapy in metastatic PCa. Furthermore, it was demonstrated in 236 PCa patients that HER2 over expression is associated with a low expression of the tumor suppressor gen PTEN (phosphatase and tensin homologue) and reduced the cancer-specific survival [55]. Using the AdnaTest ProstateCancerSelect/Detect kit for CTC isolation from the PCa patients in their study, they captured CTCs with the EpCAM and HER2 protein [54]. The same kit was used for the analysis by Antonarakis et al. [56]. This group detected AR splice variant 7 mRNA (AR-V7) in the CTCs from patients with castration-resistant PCa. The CTCs also express HER2 and AR-V7. This variant of the AR in CTCs has no ligand-binding domain, but via an active HER2 signaling it can bypass the androgen signaling pathway. In a recently published study, the expressions of AR-V7 and PTEN were determined in CTC. The authors demonstrated that more than two PTEN negative CTCs were associated with a 3.96 hazard ratio for progression or death compared with CRPC patients with less than two PTEN negative CTCs. Moreover, a high CTC AR-V7 positive count (0–20) was associated with a radiographic progression-free survival in ezalutamid-treated patients [57].

We determined the CD44 expression in the serum, but the median concentration in the mCRPC group was only slightly decreased compared with that in the PCa-l patients. However, the concentration range was much wider in this group than in the PCa-l group. Nevertheless, we could not observe any increase in the CD44 concentration after chemotherapy in the mCRPC group. Some patients demonstrated constant levels, and others had decreased levels after chemotherapy. Ma et al. [25] showed the interaction between CD44 and HER2 in PCa cell line, and linked this relationship to potential radio resistance PCa.

In this study, we showed that the CTC count determined with the CellSearch^®^ system (CTC_CS) is more suitable for mCRPC patients than CellCollector, an in vivo isolation system. We identified a moderate correlation between the CTC counts and the biomarkers sHER2 and DKK-1, and a strong correlation with the PSA level. Additionally, we found that a CTC_CS count ≥5 cells and a PSA level >53 ng/mL presented approximately the same diagnostic potency with regard to the sensitivity and specificity for OS in our mCRPC patients. Furthermore, for better personalized characterization, it is crucial to expand the research focused on CTC phenotyping, and the interactions of these cells with coexisting, tumor-associated blood-released factors.

The limitations of our preliminary investigations are of course the small number of patients and the heterogeneous group of mCRPC patients (first and second line of chemotherapy). Likewise, the CTC platforms used here capture CTCs with an EpCam antibody but not CTCs with a mesenchymal phenotype. Moreover, we included no independent cohorts such as age match healthy woman or man. A wider characterization might provide additional information about the association between CTC and other biomarkers [13]. Lager studies are needed to further validate our findings.

## Figures and Tables

**Table 1:** Study population characteristic and demographics.

Characteristics	mCRPC	PCa-l
Patient (*n*)	12	16
Median (range), years	69 (53–72)	63 (56–75)
Median (range) BMI	27.5 (20.8–39)	29.7 (22.5–34.5)
Median PSA (range), ng/mL at baseline	25.6 (35–1200)	8.2 (0.64–38.8)
Median PSA (range), ng/mL at the last visit	44.95 (0.04–903)	0,04 (0.04–0.06)
Median CRP (range), mg/mL	7.3 (1.8–94.8)	2.3 (1–26.2)
at baseline		
Median HB (range), nmol/L	7 (6.3–9.5)	9.4 (7.9–10.5)
at baseline		
Gleason sum, n (%)		
≤7	2 (16.67)	11 (64.7)
>7	10 (83.33)	6 (35.3)
Sites of metastasis, *n* (%)		
Bone	12 (100)	
Visceral	4 (33.3)	
Nodal	10 (83.3)	
Prior treatments, n (%)		
TURP	5 (41.7)	
Androgen treatment	12 (100)	
Radiation	9 (75)	
Treatments between baseline and study end, *n* (%)		
TURP		
Surgery (RP)		14 (82.3)
HIFU		2 (11.8)
Radiation	10 (83.3)	
Bone-targeted therapy	12 (100)	
Chemotherapy		
Docetaxel	10 (83.3)	
Cabazitaxel	3 * (25)	

RP—radical prostatectomy; HB—hemoglobin; PSA—prostate-specific antigen; TURP—transurethral resection of the prostate; HIFU—high-intensity focused ultrasound; BMI—body mass index; CRP—C-reactive protein. 3 * one CRPC patient received docetaxel and switched to cabazitaxel during the study period.

**Table 2:** Serum levels of different biomarkers.

Median (Range)	mCRPC V1–V6	PCa-l	V1	*p-*Value
		V2 + V3		
CD44 (pg/mL)	710 (205.9–4878)	777.1 (230.6–3382)	783.6 (386–2440)	0.70
DKK-1 (pg/mL)	4625 (566.9–8878)	3939 (1632–10937)	3976 (1273–7988)	0.80
sHer2 (ng/mL)	3.3 (0.83–16.46)	3.3 (1.1–7.7)	3.5 (1.27–8.4)	0.39
IL-6 (pg/mL)	11.7 (1.91–180)	5.6 (1.5–587.2)	8.2 (1.0–589)	0.24
IL-8 (pg/mL)	20 (1.98–112.7)	10.8 (4.8–1127)	13.2 (2.6–1216)	0.27
CTC_CC	4 (0–820)	0 (0–5)	0 (0–9)	<0.0001
CTC_CS	8.5 (0–1428)	0 (0–1)	0	<0.0001
PSA (ng/mL)	18.5 (1–1120)	8.2 (0.64–38.8)	0.04 (0.04–1.12)	<0.0001
CRP (ng/mL)	7.3 (1.8–94.8)	2.1 (1–26.4)	n.d.	0.03

CD44—cluster of differentiation 44; DKK-1—Dickkopf1; sHER2—soluble human epidermal growth factor receptor 2; IL-6, -8—interleukin-6, -8; CTC_CC—determined with CellCollector^®^; CTC_CS—determined with the CellSearch^®^ system; PSA—prostate-specific antigen; CRP—C-reactive protein.

## Supplementary Materials

The following are available online at https://www.mdpi.com/article/10.3390/life11070664/s1, Figure S1: Heatmap of non-significant correlation coefficients (Spearman) among biomarkers of localized prostate cancer patients.
