# Supplementary material for: Association of Circulating Tumor Cells with Inflammatory and Biomarkers in the Blood of Patients with Metastatic Castration-Resistant Prostate Cancer
Source: Life (Basel). 2021 Jul 6;11(7):664. doi: 10.3390/life11070664 (PMC8307979; doi:10.3390/life11070664)
Supplement: Supplementary file 1 [file life-11-00664-s001.zip › life-1254055-supplementary.pdf]

life

MDPI

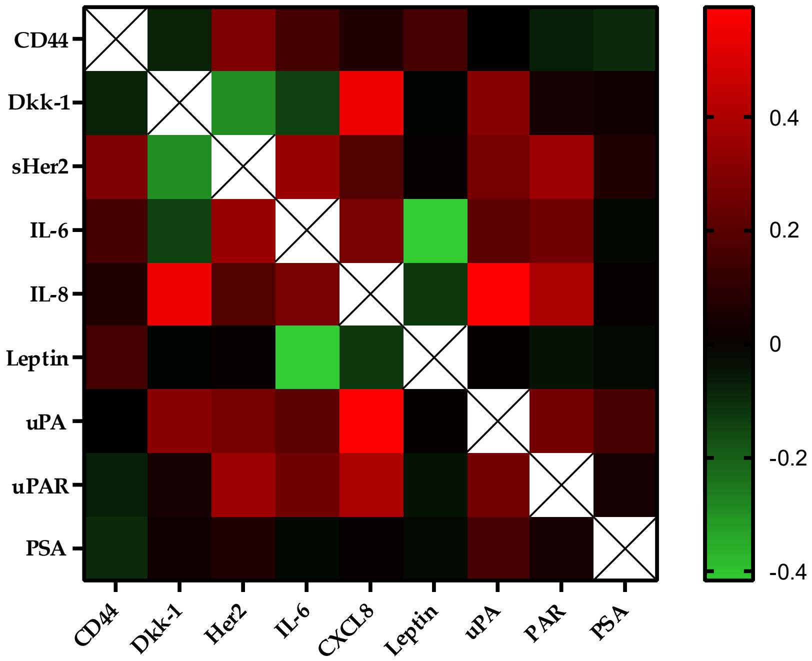

Figure S1. Heatmap of non-significant correlation coefficients (Spearman) among biomarkers of localized prostate cancer patients. The color-coded correlation is on the left, where red demonstrates a strong positive correlation and light green indicates a strong negative correlation.
